# Frequency of Circulating Regulatory T Cells Increases during Chronic HIV Infection and Is Largely Controlled by Highly Active Antiretroviral Therapy

**DOI:** 10.1371/journal.pone.0028118

**Published:** 2011-12-05

**Authors:** Pietro Presicce, Kris Orsborn, Eileen King, Jesse Pratt, Carl J. Fichtenbaum, Claire A. Chougnet

**Affiliations:** 1 Division of Molecular Immunology, Cincinnati Children's Hospital Research Foundation, Department of Pediatrics, University of Cincinnati College of Medicine, Cincinnati, Ohio, United States of America; 2 Division of Biostatistics and Epidemiology, Cincinnati Children's Hospital Medical Center, Cincinnati, Ohio, United States of America; 3 Division of Infectious Diseases, Department of Medicine, University of Cincinnati College of Medicine, Cincinnati, Ohio, United States of America; National Institute of Allergy and Infectious Diseases, United States of America

## Abstract

Regulatory T cells (Tregs) act by suppressing the activation and effector functions of innate and adaptive immune responses. HIV infection impacts Treg proportion and phenotype, although discrepant results have been reported depending on the patient population and the way Tregs were characterized. The effects of highly active antiretroviral therapy (HAART) on Treg frequency have not been thoroughly documented. We performed a detailed longitudinal analysis of Treg frequency and phenotype in 11 HIV-infected individuals enrolled in a single, prospective clinical trial, in which all patients underwent the same treatment protocol and were sampled at the same time points. Tregs were characterized for their expression of molecules associated with activation, cell cycle, apoptosis, or function, and compared to circulating Tregs from a group of age-matched healthy individuals.

Our results revealed increased proportions, but reduced absolute numbers of circulating CD3^+^CD4^+^FOXP3^+^ Tregs in chronically infected HIV-infected patients. Treg frequency was largely normalized by HAART. Importantly, we show that similar conclusions were drawn regardless of the combination of markers used to define Tregs. Our results also showed increased expression of cell cycle markers (Ki67 and cyclin B) in Tregs from untreated infected individuals, which were decreased by HAART. However, the Treg phenotype in untreated patients was not consistent with a higher level of generalized activation, as they expressed very low levels of CD69, slightly elevated levels of HLA-DR and similar levels of GARP compared to Tregs from uninfected donors. Moreover, none of these markers was significantly changed by HAART. Treg expression of CTLA-4 and cytotoxic molecules was identical between patients and controls. The most striking difference in terms of functional molecules was the high expression of CD39 by Tregs in untreated patients, which HAART only partially controlled.

## Introduction

Regulatory T cells (Tregs) act by suppressing the activation and effector functions of innate and adaptive immune responses (reviewed in [1], [2], [3]). Originally described in murine models as a subset of T cells constitutively expressing CD25, the discovery that FOXP3, a transcription factor from the forkhead box family, was necessary for Treg generation and function has allowed a better characterization of Tregs, and to date, FoxP3 remains the best marker to characterize Tregs [4], [5]. In mice and humans, mutations in *FoxP3* cause an early and fast-progressing multi-organ autoimmune disease [6]. FoxP3 is important to control immune homeostasis throughout life, as demonstrated by the uncontrolled T cell activation and rapid death following FoxP3 deletion in adult mice [7].

During chronic HIV infection, the role of Tregs is complex. On one hand, Tregs control *in vitro* HIV replication in several cellular targets and protect host tissues from immune-mediated damage [8], [9]. On the other hand, Tregs dampen HIV-specific T cell responses, and they might thus facilitate the establishment and maintenance of a chronic infection (rev. in [10], [11]. However, transient depletion of CD25^+^ T cells in chronically SIV-infected African green monkeys triggered increases in immune activation and viral replication and depletion of mucosal CD4^+^ T cells [12], suggesting that Tregs can have both detrimental and beneficial roles during HIV infection.

HIV infection impacts Treg frequency and phenotype, although discrepant results have been reported depending on the patient population and the way Tregs were characterized. Several studies described increased percentage of Tregs in the circulating blood of chronically infected individuals compared to healthy controls or long-term non-progressors, although absolute numbers of Tregs were decreased [13]–[23]. However, other studies reported decreased FOXP3 mRNA in untreated patients [13], [14], decreased percentage of CD4^+^CD25^+^CD127^−^FOXP3^+^ Tregs in African HIV-1 infected subjects [15] or decreased percentage of FOXP3^+^ cells in the CD25^bright^ subset of CD4^+^ T cells [16]. The effect of HAART on Treg frequency has not been clearly established. In addition to the variables noted above, other inconsistencies complicate this type of analysis: patients participating in clinical research studies are often treated by different antiretroviral regimens; specimens may be collected at few, and inconsistent, time points in the longitudinal studies; some studies may be cross-sectional rather than longitudinal. To overcome these limitations, we performed a detailed longitudinal analysis of Treg percentage and phenotype in individuals enrolled in a single, prospective clinical trial, which allowed us to eliminate variability in terms of treatment and time points post HAART initiation. We also examined whether the combination of markers used to define Tregs would influence the interpretation and conclusions. In addition, Tregs were characterized for their expression of molecules associated with activation, cell cycle, apoptosis or function.

## Results

### Subject description and HAART efficiency

Eleven adult patients chronically infected with HIV-1 were co-enrolled in our study and in a clinical trial of tenofovir/emitricitabine plus lopinavir/ritonavir. At baseline, the patients' median peripheral CD4 count was 288 cells/µL (range: 20–615 cells/µL) and the median viral load was 48,763 copies/mL (range: 4,400–750,000 copies/mL). Samples were collected before treatment (wk0), at wk2, wk4, wk8 and wk24 post treatment initiation, as well as after a minimum of 46 weeks of HAART (wk46+). In parallel, we enrolled a group of age-matched HIV-uninfected healthy control subjects. Demographic information of the patients and controls are summarized in Table 1.

**Table 1:** Baseline characteristics and treatment information.

Variable	Category	HIV-infected (n = 11)^a^	Control (n = 12)^a^
Age		40 (37–49)	39.5 (30–53)
Gender	Male/Female	11/0	10/2
Race	White/African American	5/6	8/4
CD4 counts (cells/µl)		288 (20–615)	951 (628–1716)
%CD4^+^		16 (4–31)	50 (42–66)
Viral load (copies/mL)		48,763 (4,400–750,000)	N/A
Hepatitis C	Positive/negative	1/10^b^	
Hepatitis B	Positive/negative	0/11^b^	
Treatment naïve		27.2%^c^	
ARV	TDF/FTC+LPV/RTV	100%^c^	
Adherence score	Wk4	4 (3–4)^d^	
	Wk8	4 (3–4)^d^	
	Wk24	4 (1–4)^d^	
	Wk46+	4 (1–4)^d^	

a : Values are expressed in median (range) or ratio for numerical values and categorical values respectively.

b : One subject had antibodies to hepatitis C virus;

c : indicates the proportion of enrolled patients with the described characteristic;

d : median (range) adherence score, on a scale of poor (1) to excellent (4), at each time point. TDF/FTC-tenofovir/emitricitabine; LPV/RTV-lopinavir/ritonavir.

**Figure 1:**
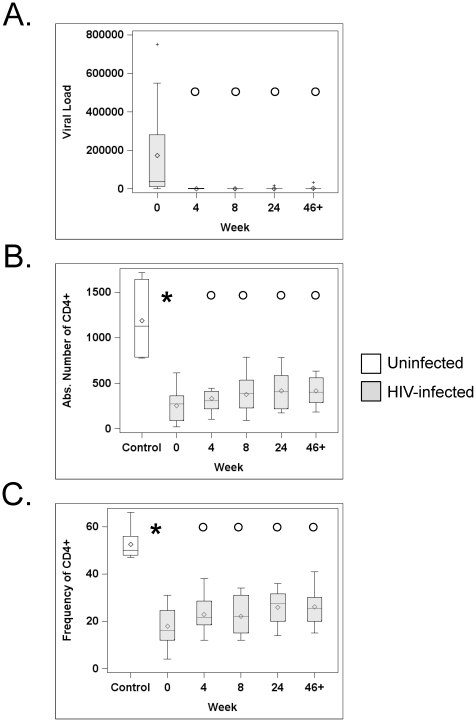
HAART decreases Viral Load and increases CD4 counts and percentages. Viral loads, expressed in RNA copies/ml (A), absolute CD4 counts, expressed in cells/ul (B) and CD4^+^ T cell frequency, expressed in % (C), were analyzed longitudinally in 11 HIV-infected patients starting HAART (grey boxes). Absolute CD4 counts and CD4 T cell frequency were also analyzed in 8 age-matched healthy controls (white boxes, panel B and C). The horizontal bars inside the box correspond to the median (50^th^ percentile), the rhombus correspond to the mean, the box limits correspond to the 25th and 75th percentiles, and the vertical bars correspond to the 5th and 95th percentiles. Outliers are indicated by a + sign. An asterisk (*) indicates a significant difference between controls and HIV-infected subjects at baseline (wk0). A circle (°) indicates a significant difference between wk0 and the indicated time point after HAART initiation in HIV-infected subjects.

All but one patient had an appropriate response to the drug regimen during the follow-up with excellent self-reported adherence and pill counts (median adherence score of 4, on a 1–4 scale, at all time points, see Table 1). The non-compliant patient stopped HAART between wk24 and wk46+. As expected, median virus load (VL) dropped rapidly after patients started treatment and was significantly lower at all time points than at baseline (all p<0.0001, Fig. 1A). At wk24, 9 of 11 patients had undetectable VL (<50 copies/mL). HIV was undetectable in 7 of the 10 patients still under treatment at the latest time point (>46 weeks), and viral loads remained low (<1,300 copies/mL) in the 3 other treated patients. Median absolute numbers and frequency of CD4^+^ T cells increased at all time points compared to baseline (Fig. 1B and 1C).

### Treg frequency is increased in HIV-infected patients at baseline and follows a biphasic curve after treatment initiation

**Figure 2:**
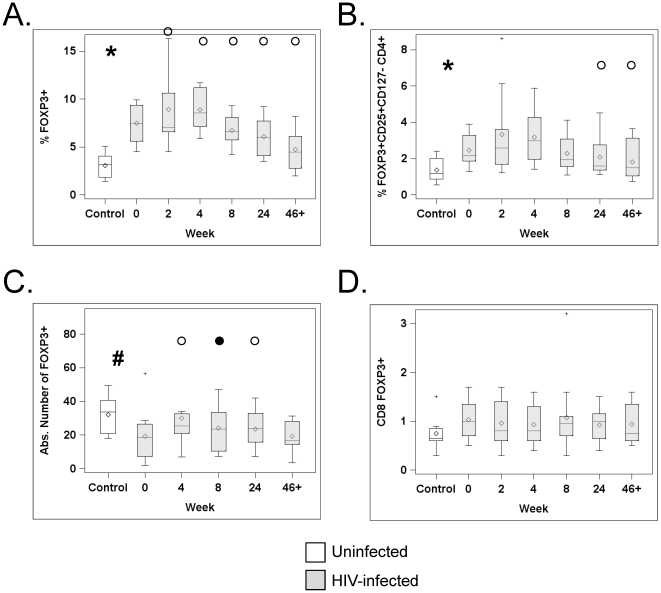
Treg frequency shows a biphasic curve post-HAART. PBMCs from HIV-uninfected subjects (white boxes) and HIV-infected subjects at different time points after HAART initiation (grey boxes) were analyzed by flow cytometry. Graphs show in (A): the percentage of FOXP3^+^ cells in CD3^+^CD4^+^ T cells; (B) the percentage of FOXP3^+^CD25^+^CD127^lo^ cells in CD3^+^CD4^+^ T cells; (C) the absolute numbers of CD3^+^CD4^+^FOXP3^+^ cells (expressed in cells/µL); and (D) the frequency of FOXP3^+^ cells in CD3^+^CD8^+^ lymphocytes. The horizontal bars inside the box correspond to the median (50th percentile), the box limits correspond to the 25th and 75th percentiles and the vertical bars correspond to the 5th and 95th percentiles. Outliers are indicated by a + sign. An asterisk (*) indicates a significant difference (p≤0.05), and a pound sign (#) indicates a trend (p≤0.10), between control and infected subjects at baseline (wk0). An open circle (°) indicates a significant difference (p≤0.05), and a close circle (•) indicates a trend (p≤0.10) between baseline (wk0) and the indicated time point after HAART initiation in HIV-infected subjects.

Treg were first defined as CD3^+^CD4^+^FOXP3^+^ small lymphocytes. A representative example of flow cytometry analysis is shown in Figure S1. As shown in Figure 2A, the baseline frequency of FOXP3^+^ cells was increased in HIV-infected patients by ∼2-fold compared to controls (median: 7.9% and 3.15% of CD3^+^CD4^+^, respectively, p = 0.0004, Wilcoxon Rank Sum test). As the usage of different anti-FOXP3 clones has previously led to discrepant data, we then defined FOXP3^+^ cells as double positive for staining with two anti-FOXP3 clones (PCH101 and 259D). As expected [17], the proportions of Tregs defined that way were lower in both subject groups than if defined as PCH101^+^ only (1.6% in untreated patients versus 1% in controls), but differences between groups showed similar trends, although they did not reach statistical significance (p = 0.14). For the rest of the studies, we only used the anti-FOXP3 clone PCH101.

In HIV-infected patients, baseline CD4^+^FOXP3^+^ frequency was not correlated with their CD4 counts (r = −0.036, p = 0.92), although there was a trend towards a correlation with their viral loads (r = 0.496, p = 0.12, Spearman test). Within FOXP3^+^CD4^+^ T cells, levels of FOXP3 were not different in the 2 groups (geometric mean FOXP3 MFI of 2847 and 2759 for patients and controls, respectively, n = 9/group, p = 0.91, Wilcoxon signed rank).

Treg percentage was assessed longitudinally after patients started HAART. Interestingly, this parameter followed a biphasic curve, increasing in the first weeks (geometric mean relative increases of +20% and +17% at wk2 and wk4, both p<0.035), but then dropping at wk8 and continuing a downward trajectory in the following weeks (geometric mean relative decreases versus baseline of −13%, −24% and −43% at wk8, wk24 and wk46+ respectively; p = 0.05, 0.0004 and <0.0001, Fig. 2A). By >46 weeks of HAART, Treg frequency was largely normalized, as the difference with control was not statistically significant (median: 3.2% for control, 4.2% for patients at wk46+, p = 0.11 Wilcoxon Rank Sum). FOXP3 levels per cell were not changed by HAART (all p>0.10 in comparison to wk0, repeated measures ANOVA).

Human Tregs have also been identified as CD3^+^CD4^+^FOXP3^+^CD25^+^CD127^lo^, although this more stringent definition typically underestimates the total number of Tregs [17]. Using this combination of markers, lower proportions of Tregs were found in both groups, but overall comparisons led to similar results: untreated HIV-infected patients exhibited a 2-fold increase in the frequency of FOXP3^+^CD25^+^CD127^lo^ CD4^+^ T cells compared to controls (median of 2.3% versus 1.2%, p = 0.013, Fig. 2B). Following HAART initiation, this gating strategy revealed a trajectory similar to CD3^+^CD4^+^FOXP3^+^: the proportion of CD4^+^ T cells that were FOXP3^+^CD25^+^CD127^lo^ first increased at wk2 and wk4, although the difference with wk0 was not significant. Thereafter, the FOXP3^+^CD25^+^CD127^lo^ population declined, becoming significantly less frequent than baseline at wk24 and wk46+ (Fig. 2B).

Based on these data, we defined Tregs as CD3^+^CD4^+^FOXP3^+^ (PCH101^+^) cells for the rest of the analyses. We also determined absolute numbers of CD4^+^FOXP3^+^ T cells at baseline and after treatment (Fig. 2C). Due to the low absolute CD4 counts in patients, absolute numbers of CD4^+^FOXP3^+^ were decreased at baseline in these patients compared to healthy controls, although the difference did not reach statistical significance (p = 0.09). Mirroring the increases in total CD4 counts occurring after HAART initiation, absolute numbers of CD3^+^CD4^+^FOXP3^+^ significantly increased at wk4 and wk24, compared to wk0 (p = 0.009, 0.05, respectively). At wk8, the numbers also increased but this trend did not reach statistical significance (p = 0.06).

As noted above, only a proportion of FOXP3^+^CD4^+^ T cells also expressed CD25, but this percentage was similar in HIV-infected patients at baseline and healthy controls (median: 36.8% versus 32.9%, p = 0.87), and was not affected by HAART (data not shown).

### Frequency of CD8^+^FOXP3^+^ is low and unchanged by HIV infection

Expansion of CD8^+^FOXP3^+^ T cells has been described during the acute infection of rhesus macaques [18] as well as in AIDS patients [19]. Low frequencies of these cells were found in HIV-infected and uninfected subjects and were not statistically different (median of 1% and 0.65% of total CD8^+^ T cells in patients at baseline and controls, respectively, p = 0.14, Fig. 2D), and HAART did not affect this cellular subset (all p>0.37, in comparison to wk0).

### Expression of cell cycle markers is increased in Tregs from HIV-infected patients

**Figure 3:**
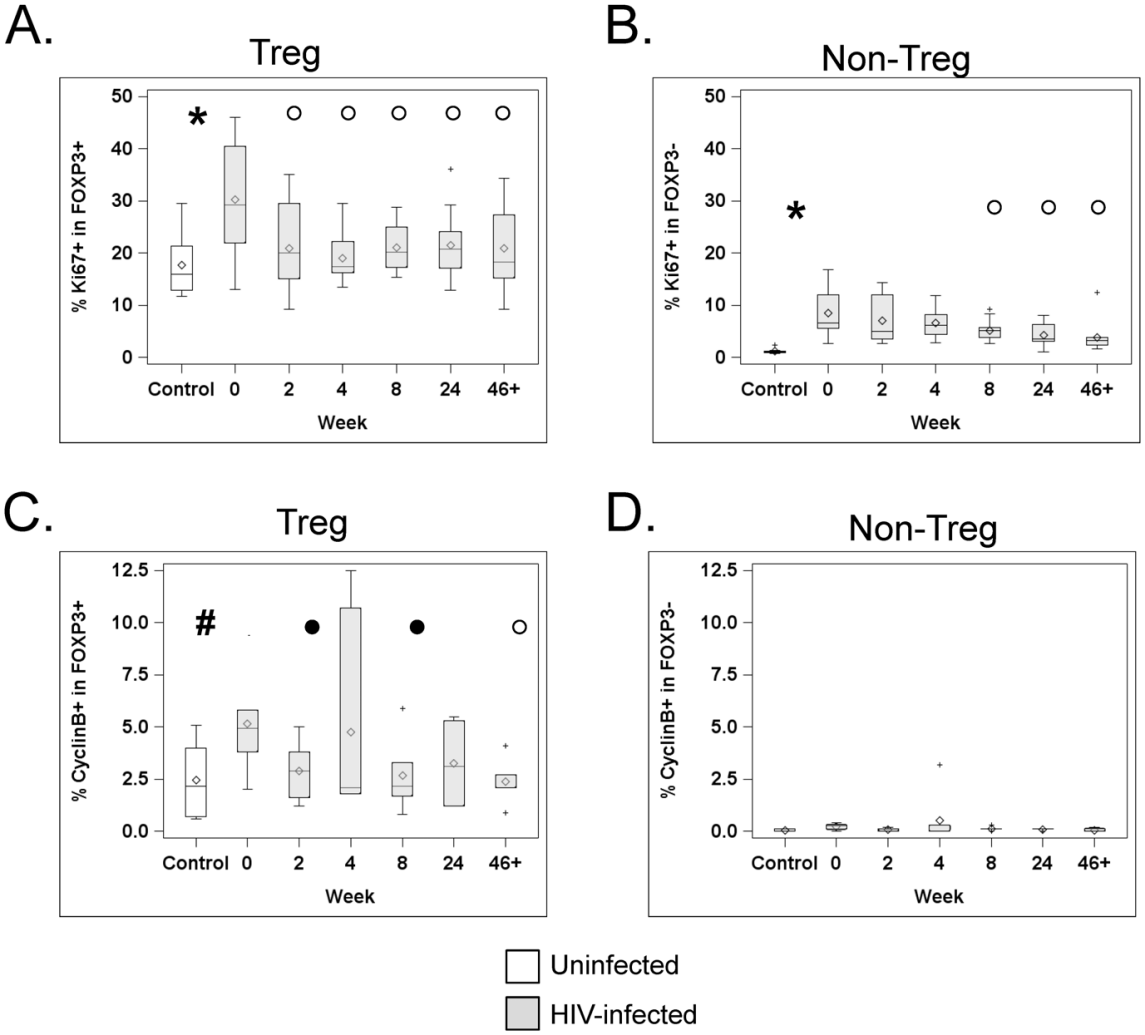
HAART decreased enhanced expression of cell cycling markers in Tregs from HIV-infected subjects. PBMC from HIV-uninfected subjects (white boxes) and HIV-infected subjects at different time points after HAART initiation (grey boxes) were analyzed by flow cytometry. Graphs show the percentage of Ki-67^+^ (A) Tregs (defined as CD3^+^CD4^+^FOXP3^+^ cells) and (B) non-Tregs (defined as CD3^+^CD4^+^FOXP3^−^ cells), as well as the percentage of Cyclin B^+^ cells in Tregs (C) and non-Tregs (D). The horizontal bars inside the box correspond to the median (50th percentile), the box limits correspond to the 25th and 75th percentiles and the vertical bars correspond to the 5th and 95th percentiles. Outliers are indicated by a + sign. An asterisk (*) indicates a significant difference (p≤0.05), and a pound sign (#) indicates a trend (p≤0.10), between control and infected subjects at baseline (wk0). An open circle (°) indicates a significant difference (p≤0.05), and a close circle (•) indicates a trend (p≤0.10) between wk0 and the indicated time point after HAART initiation in HIV-infected subjects.

We next characterized whether increased Treg frequency in HIV infection was associated with enhanced cell cycling in these Tregs. To do this, we looked at levels of Ki67, which is expressed during all active phases of the cell cycle (G_1_ to mitosis), but is absent from resting cells (G_0_). Interestingly, Tregs expressed Ki67 more often than non-Tregs in control subjects (median 16% versus 1%, p = 0.008, Wilcoxon signed ranked test, see Fig. 3A for Treg and Fig. 3B for non-Tregs). In infected patients, Ki67 expression was increased in both Tregs and non-Tregs at baseline, compared to uninfected donors: the median percentage of Ki67^+^ Tregs was 27.2% in HIV-infected patients versus 16% in uninfected subjects (p = 0.006; Fig. 3A); the frequency of Ki67^+^ non-Tregs was 6.6% in patients versus 1% in controls (p = 0.0003, Fig. 3B). At wk0, the percentage of Ki67^+^ Tregs was directly correlated with patients' VL (r = 0.709, p = 0.018, Spearman correlation) and inversely correlated with patients' CD4 counts (r = −0.873, p = 0.0009). However, it did not correlate with the overall Treg frequency or the activation of non-Tregs, as evidenced by their expression of Ki67 or HLA-DR (all p>0.12, Spearman correlations).

HAART significantly decreased Ki67 expression in Tregs (all p<0.04 versus wk0). Levels dropped very early (by wk2) and stayed constant until wk46+ (p = 0.78, repeated measures ANOVA comparison of wk2 through wk46+, Fig. 3A). As expected based on previous studies showing that HAART reduces immune activation (reviewed in [20]), Ki67 expression in non-Tregs also decreased after treatment, but the decline was more progressive than that occurring in Tregs, as Ki67 levels were significantly decreased versus wk0 levels only after 8 weeks of HAART (difference versus wk0: p>0.05 at wk 2 and 4, but p<0.005 at wk8, wk24 and wk46+, Fig. 2B).

To confirm these data, we assessed the expression of cyclin B, which controls cell cycling at the G2 phase to mitosis transition, in 6 of the 11 patients and 6 controls. As expected due to its more restricted temporal expression, the proportion of cells expressing cyclin B was much lower than that of Ki67^+^ cells (see Fig. 3A and 3C), but equivalent trends were observed, with a trend towards higher percentage for Tregs from HIV-infected patients than those of uninfected donors (median % of cyclin B^+^ Tregs: 5.0% and 2.2% in HIV-infected and uninfected subjects, respectively, p = 0.06, Fig. 3C). Similar tendency was seen in non-Tregs, although differences were not significant (median % of cyclin B^+^ non-Tregs: 0.25% and 0.0% in HIV-infected and uninfected subjects, respectively, p = 0.12, Fig. 3D). Expression of cyclin B in Tregs decreased after HAART, and wk46+ values were significantly lower than wk0 values (Fig. 3C).

### Expression of Bim and Bcl-2 by Treg is not affected by HIV infection

The balance of pro- and anti-apoptotic molecules results in cell death or survival, and apoptotic pathways are an important rheostat of circulating cell frequency. The pro-apoptotic Bim and the anti-apoptotic Bcl-2 are particularly critical for the regulation of T cell survival [21]. We thus measured the expression of these molecules in Tregs during HIV infection. Because Bim and Bcl-2 are differently expressed by naïve and memory T cells [21] and the proportion of naïve/memory Tregs varies between individuals, we analyzed their levels in gated naïve and memory Tregs (CD45RA^+^ or CD45RO^+^ FOXP3^+^), in a subset of 7 out 11 patients and 7 matched controls. Due to the large intra-experiment variability of MFIs, samples collected longitudinally from each HIV-infected individual were analyzed in the same experiment, along with the cells from one healthy control. Statistical comparisons were done using the Wilcoxon Signed Rank test, matching infected patients with the controls analyzed the same day.

Equivalent levels of expression of Bim or Bcl-2 were found in Tregs between controls and untreated HIV-infected subjects (all p>0.15), except for a trend toward higher Bcl-2 levels in naïve Tregs in HIV-infected patients (p = 0.08). In general, HAART did not affect the levels of Bim or Bcl-2 in Tregs, except for a marginal and isolated decrease in Bim expression in memory Tregs at wk46+ compared to wk0 (p = 0.024).

Of note, percentages of naïve and memory Tregs were similar in HIV-infected individuals and healthy controls (both p>0.30). Increased frequency of naïve Tregs was found at wk46+ (geometric mean relative change of +6.7 fold, p = 0.002).

### Tregs from HIV-infected patients express normal levels of CTLA-4 and cytotoxic molecules, but increased levels of CD39

Tregs dispose of a variety of immune suppressive mechanisms and they appear to exert their function differently depending on the tissue, the inflammatory milieu, and what triggered the immune response (for review, see [1]–[3]). Several molecules are strongly associated with Treg function, and we therefore characterized their expression.

**Figure 4:**
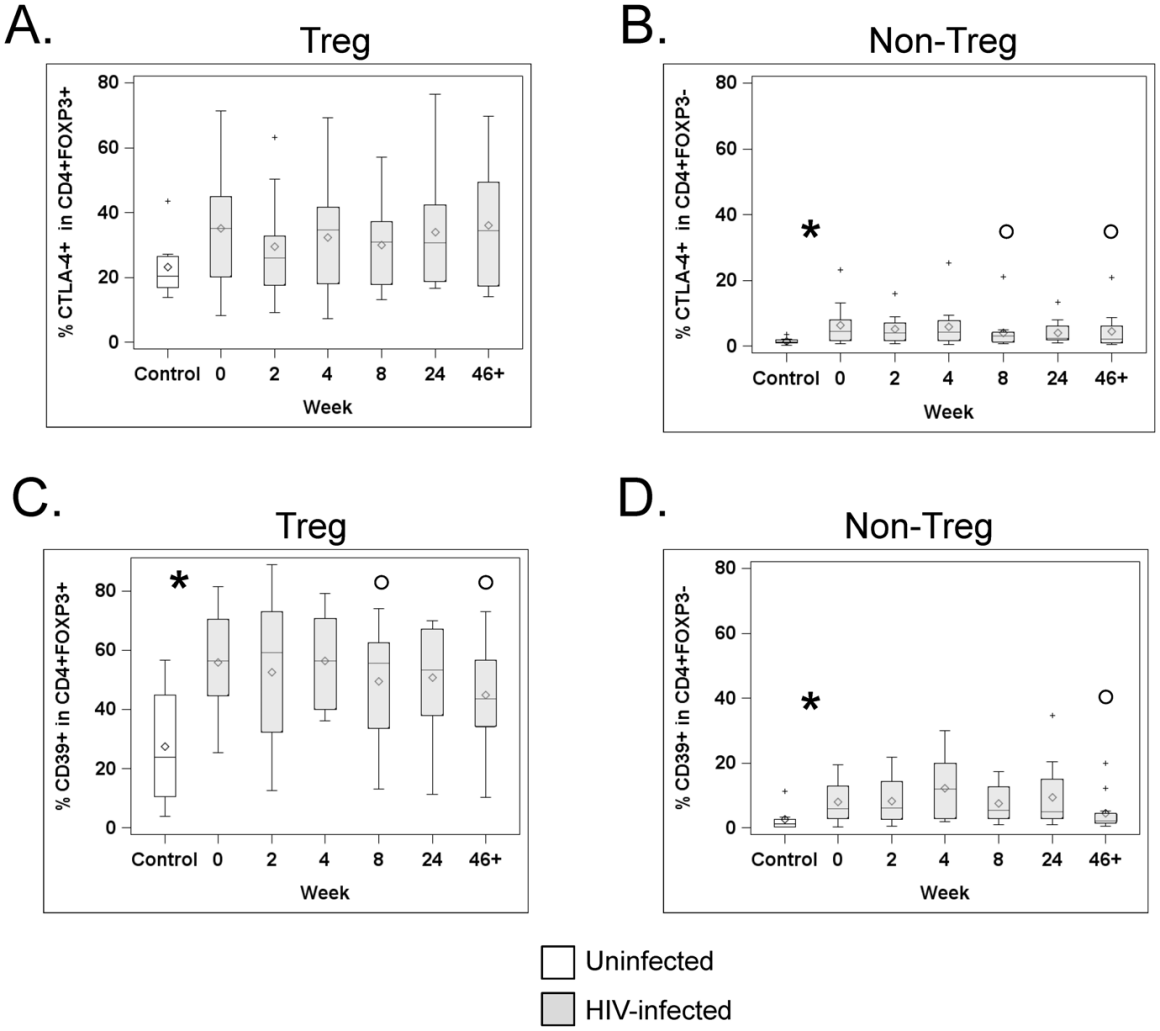
HAART does not affect CTLA-4 expression, but significantly decreases CD39 expression in Tregs. PBMCs from HIV-uninfected subjects (white boxes) and HIV-infected patients at different time points after HAART initiation (grey boxes) were analyzed by flow cytometry. Graphs show the percentage of CTLA-4^+^ (A) Tregs (defined as CD3^+^CD4^+^FOXP3^+^ cells) and (B) non-Tregs (defined as CD3^+^CD4^+^FOXP3^−^ cells), as well as the percentage of CD39^+^ cells in Tregs (C) and non-Tregs (D). The horizontal bars inside the box correspond to the median (50th percentile), the box limits correspond to the 25th and 75th percentiles and the vertical bars correspond to the 5th and 95th percentiles. Outliers are indicated by a + sign. An asterisk (*) indicates a significant difference (p≤0.05), and a pound sign (#) indicates a trend (p≤0.10), between control and infected individuals at wk0. An open circle (°) indicates a significant difference (p≤0.05), and a close circle (•) indicates a trend (p≤0.10) between wk0 and the indicated time point after HAART initiation in HIV-infected subjects.

CTLA-4 is an essential mediator of Treg function, as shown in mice lacking CTLA-4 specifically in Tregs [22]. Confirming previous results, CTLA-4 was mainly expressed by Tregs: 35.1% of Tregs expressed CTLA-4 vs. 3.2% of non-Tregs in HIV-infected patients at wk0 (see Fig. 4A and Fig. 4B; p<0.008, Wilcoxon signed rank test); equivalent difference in CTLA-4 expression was found between Tregs and non-Tregs of controls (20.5% versus 1.2%, p<0.008). CTLA-4 expression by Tregs was not different between controls and patients at baseline (p = 0.23, Fig. 4A), and HAART did not change these levels (all p>0.17 versus wk0). In contrast to Tregs, CD4^+^ non-Tregs from untreated HIV-infected patients displayed increased CTLA-4 expression compared to controls (p = 0.02, Fig. 4B), as previously reported [23], although it remained much lower than that of Tregs. This proportion was significantly decreased by HAART, although not at all time points (p<0.05 at wk8 and wk46+).

Cytotoxic molecules, particularly granzyme A and perforin, are upregulated on the surface of activated human Tregs, and these activated Tregs can display perforin-dependent cytotoxicity against autologous target cells [24]. We therefore measured granzyme A and perforin expression by Tregs. Both molecules could be found on a subset of Tregs, with slightly higher levels in Tregs from HIV-infected patients than in healthy controls, but differences were not significant (both p>0.34, Table 2). HAART did not affect the expression of these molecules (all p>0.11 versus wk0).

**Table 2:** Expression of granzyme A and perforin by Tregs.

	Gran A	Perforin
*Median at baseline* a		
Controls	5.8 (3.1–27.5)	1.2 (0.7–8.8)
HIV-infected (wk0)	10.8 (0.7–22.0)	3.5 (0.2–19.9)
*Changes after HAART* b		
Wk2	−38.7 (95)	−45.7 (185)
Wk4	−32.3 (95)	−52.8 (185)
Wk8	−21.3 (99)	−27.4 (193)
Wk24	+9.4 (99)	+28.4 (193)
Wk46+	+50.7 (99)	+44.8 (193)

a : data are shown as median (range) percentage of Tregs expressing each marker. Markers were measured in 11 HIV-infected patients at wk0, and in 8 or 7 controls, for granzyme A and perforin respectively. None of these percentages were significantly different.

b : data are shown as relative changes in geometric means, expressed in % (coefficient of variation). Formulas used to calculate these values are as follows: relative change in geometric means = 100*[geometric mean (post HAART/wk0)]-100; coefficient of variation = [exp(standard deviation on log scale)-1]*100. Granzyme and perforin levels at all time points post HAART were similar to wk0 values (all p>0.11, repeated measures ANOVA).

Several studies have recently reported that Tregs constitutively express the ectonuclease CD39, and this pathway appears critical for Treg function [25], [26]. In agreement with these previous studies, in healthy controls, CD39 was found mainly expressed by Tregs, as 23.7% of Tregs expressed CD39 versus 1.2% of non-Tregs (see Fig. 4C and 4D, p = 0.008, Wilcoxon signed ranked test). Interestingly, the proportion of Tregs expressing CD39 was elevated in HIV-infected individuals at baseline compared to controls (59% versus 23.7%, p = 0.005, Fig. 4C), although this percentage was not correlated with the patients' VL (r = −0.018, p = 0.96, Spearman correlation) or CD4 counts (r = −0.109, p = 0.75), or Treg frequency (r = −0.055. p = 0.88). This percentage also did not correlate with the level of activation of non-Tregs (as evidenced by their expression of Ki67 or HLA-DR, both p>0.35). The percentage of CD39^+^ non-Tregs was also elevated in patients, but its expression remained confined to a minority of these cells (4.5% versus 1.2%, p = 0.02, Fig. 4D). After ≥46 weeks of HAART, expression of CD39 by Tregs and non-Tregs was significantly decreased (Fig. 4C and 4D), but the percentage of CD39^+^ Tregs tended to remain higher than in healthy controls, although the difference did not reach statistical significance (median: 48.3 vs 23.7%, p = 0.07, Wilcoxon Rank Sum Test).

Because CD39 and CTLA-4 are associated with Treg activation, we also measured the levels of two classical activation markers, CD69 and HLA-DR. In agreement with our previous data [27], circulating Tregs did not express CD69 (median frequency of CD69^+^ Tregs was 0.5% and 1% in untreated patients and controls respectively, p = 0.4, Table 3). In contrast, a sizable proportion of Tregs expressed HLA-DR, in both controls (median 21.2%) and HIV-infected patients (median 41.1%), and this proportion was much higher than that found for the corresponding non-Tregs (median % of HLA-DR^+^ in non-Tregs: 1.7% in controls and 12.1% in untreated HIV-infected patients). Although the frequency of HLA-DR^+^ Tregs was increased by ∼2-fold in patients compared to controls, the difference did not reach statistical significance (p = 0.10, Table 3), and HAART did not significantly alter it (all p>0.15 versus wk0, Table 3). In contrast, HLA-DR expression by non-Tregs was significantly higher in HIV-infected patients than in controls (p = 0.03, Table 3), and decreased after approximately 1 year of HAART (wk46+ vs. wk0: p = 0.02). As high levels of glycoprotein A repetitions predominant (GARP, or LRRC32) mRNA had been found in activated human Tregs [28], we also determined its expression by Tregs. The proportion of GARP^+^ Tregs was similar in controls and untreated patients (median of 11.9% vs. 14.7%, respectively, p = 0.18), and HAART did not change it (all p>0.53 vs. wk0).

**Table 3:** Expression of activation markers by Tregs and non-Tregs.

	CD69	CD69	HLA-DR	HLA-DR
	Tregs	Non-Tregs	Tregs	Non-Tregs
*Median at baseline* a				
Controls	1.05 (0.3–6.1)	0.4 (0.2–1)	21.2 (8.9–54.7)	1.8 (0.5–10.5)
HIV-infected	0.5 (0.1–23.2)	*0.9 (0.1–8.7)* *	41.1 (21.0–69.2)#	*12.1 (0.3–42.9)* *
*Changes after HAAR T* b				
Wk2	+2.0 (90)	−26.7 (85)	−20.5 (94)	−5.8 (99)
Wk4	−13.1 (96)	−22.1 (90)	+15.0 (96)	−22.1 (110)
Wk8	−4.9 (96)	−26.7 (90)	−36.9 (93)	−8.6 (102)
Wk24	+2.0 (96)	−37.5 (90)[Table-fn nt112] 	−28.8 (93)	−23.7 (102)
Wk46+	+4.1 (92)	−40.0 (86)[Table-fn nt112] 	−29.5 (93)	*−57.3 (102)* o

a : data are shown as median (range) percentage of Tregs (defined as CD3^+^CD4^+^FOXP3^+^) or non-Tregs (defined as CD3^+^CD4^+^FOXP3^−^) expressing each marker. Markers were measured at wk0 in 11 and 9 HIV-infected patients for CD69 and HLA-DR respectively, and in 8 controls for both markers.

*and italics: indicates a significant difference between controls and HIV-infected subjects (p≤0.05);

# : indicates a trend towards significance (p≤0.10).

b : data are shown as relative changes in geometric means, expressed in % (coefficient of variation). Formulas used to calculate these values are as follows: relative change in geometric means = 100*[geometric mean (post HAART/wk0)]-100; coefficient of variation = [exp(standard deviation on log scale)-1]*100. Differences versus baseline were analyzed using the repeated measures analysis of variance.

o and italics: indicates a significant difference versus wk0 (p≤0.05);

^
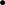
^indicates a trend towards significance (p≤0.10).

## Discussion

In the present study, we followed the frequency, absolute numbers and detailed phenotype of circulating Tregs in HIV-infected patients who were starting HAART. All patients were given the same drug regimen; adherence to treatment was carefully monitored and was in general excellent. Such experimental design allowed us to overcome the limitations of some previous studies of the effect of HAART on Tregs, as it eliminated variability in terms of treatment and time points post treatment initiation.

Our data revealed an approximate 2-fold increase in Treg frequency in untreated chronically infected patients compared to age-matched healthy controls. Importantly, similar results were obtained whether Tregs were defined simply as CD3^+^CD4^+^FOXP3^+^ or as CD3^+^CD4^+^FOXP3^+^CD25^+^CD127^lo^, which is the most stringent definition of human Tregs. Furthermore, the clone used to detect FOXP3 did not change the general conclusion. These data are in agreement with several studies that used different definitions of Tregs, such as FOXP3^+^CD25^hi^CD127^lo^ [29]–[31], FOXP3^+^ [29], [30], FOXP3^hi^CD25^hi^ [31]–[33], CD25^+^CD127^lo^ [19], [31], or CD25^hi^ [32]. Although the percentage of CD4^+^FOXP3^+^ T cells was increased, the levels of FOXP3 per cell were similar in infected patients and controls, suggesting that HIV infection does not affect FOXP3 *per se*, but affects the homeostasis of the Treg population. We and other investigators had previously reported decreased FOXP3 mRNA levels in infected patients [13], [14], and this major difference in the way FOXP3 was measured could explain the discrepant findings. However, a conflicting result comes from a study conducted in Uganda by Eller et al., who described a reduction of the CD4^+^CD25^+^CD127^−^FOXP3^+^ Treg compartment in HIV-infected subjects [15]. Reasons for this discrepancy are not clear, but could come from a different level of immune activation in Africans compared to populations residing in more developed countries.

Following HAART initiation, Treg frequency followed a biphasic curve. It first increased, before dropping after 8 weeks. A model to explain our data could be that HAART leads to an early release of Tregs from lymphoid organs where they accumulate due to the high viral loads [14], [33], explaining the initial increase, but after few weeks, the trajectory is inverted as HAART starts controlling virus replication and Treg proportion starts to normalize. Interestingly, Weiss et al. described the reverse phenomenon, as they showed a significant augmentation in Treg percentages 12 months after HAART interruption [34]. HAART influence on Treg frequency could be of great relevance to understand the pathogenesis of the immune reconstitution inflammatory syndrome (IRIS). We could not directly address this question because no patient developed IRIS in our limited cohort, as expected given the baseline median CD4 of 288 cells/µL, but similar frequencies of circulating Tregs were previously reported in patients regardless of whether or not they developed IRIS [31], [35]. Additional characterization of the functional capacity of Tregs will be required to better understand the relationship between Tregs and the development of IRIS.

Mechanisms underlying increased frequency of Tregs during HIV infection are not well understood, but could include increased proliferation and/or lower cell death. Of note, absolute numbers of Tregs are diminished during chronic HIV infection, and increased after HAART initiation, suggesting that HIV partially depletes Tregs, albeit to a lesser extent than non-Tregs. One striking result in our study is the increased level of molecules associated with cell cycling in Tregs from HIV-infected individuals. Tregs have long been described as being anergic, based on results from *in vitro* studies. *In vivo* studies have since challenged this assumption, as Tregs were shown to express high levels of cell cycle markers and to proliferate more *in vivo* than non-Tregs, in humans [36] and mice [37]. Our data are in agreement with these more recent studies, as Tregs from healthy subjects expressed Ki67 and cyclin B more often than non-Tregs. Importantly, further upregulation of the expression of cell cycle markers by Tregs occurred in untreated HIV-infected patients but was quickly controlled upon HAART initiation. Other groups have also found higher percentages of Ki67^+^ Tregs in untreated HIV-infected patients, particularly in patients with low CD4 counts [29], [30], [38], but the effect of HAART has not been consistent between studies. In agreement with our data, Xing et al. reported a reduction of Ki67 expression after 6 months of therapy in patients who responded to treatment [38]. Similarly, decreased Ki67 levels were found in a cross-sectional study of HAART responders compared to non-responders or untreated patients [30]. In contrast, Bi et al. reported very minor changes in Ki67 expression in Tregs following HAART initiation [29]. To confirm the Ki67 data, we therefore characterized the expression of a later marker of cell cycling, cyclin B, which controls cell entry into mitosis. Tregs from untreated patients also expressed cyclin B more frequently than those of controls, further supporting the hypothesis of a high Treg turnover during HIV infection. Previous studies have reported cyclin B over-expression in CD4^+^ T cells from infected patients, but they did not differentiate between Tregs and non-Tregs [39], [40]. This reported increase could thus have been due to a combination of overall increased Treg proportion in HIV-infected subjects and increased percentage of cyclin B^+^ cells, Tregs or non-Tregs. Of interest, although Tregs from HIV-infected individuals expressed cell cycle markers more often than those of healthy controls, their phenotype was not consistent with generalized higher level of activation. Indeed, at wk0, they expressed very low levels of CD69, slightly elevated levels of HLA-DR and similar levels of GARP compared with Tregs from uninfected donors. Moreover, none of these markers was significantly changed by HAART. Our data thus suggest that Treg proliferation may play a role in their increased frequency during untreated HIV infection, but this hypothesis will have to be rigorously tested in the SIV model. Mechanisms underlying increased Treg proliferation in HIV-infected patients are not known but could be multiple. HIV antigens could play a direct role, as shown *in vitro* for cyclin B upregulation [41]. Alternatively, enhanced microbial translocation could be the trigger, as a direct engagement of Treg Toll-like receptor 4 by liposaccharides induces their proliferation without the need for T cell receptor ligation [42].

Relative Treg insensitivity to death could also lead to their augmented frequency in HIV-infected patients. We therefore looked at the levels of expression of the pro-apoptotic molecule Bim and the anti-apoptotic molecule Bcl-2 by Tregs, and found no major differences between HIV-infected patients, before or after HAART, and controls. However, these negative data do not rule out improved Treg survival as a mechanism underlying increased Treg frequency during HIV infection, as suggested by *in vitro* data [33]. Moreover, tissue Tregs appear to be less frequently productively infected than non-Tregs in SIV-infected Rhesus macaques [43], [44], a finding supported by our *in vitro* data [45], and this difference could lead to selectively higher Treg survival. Of note, we checked the percentage of HIV Gag^p24+^ CD4^+^ T cells (Tregs and non-Tregs) at wk0 in our patients, but these percentages were too low to draw clear conclusions (data not shown).

Expression of several functional molecules by Tregs was either identical in patients and controls (CTLA-4 or cytotoxic molecules), or increased in patients (CD39). These data are in agreement with the fact that, in general, Tregs have been found to be suppressive in HIV-infected patients or SIV-infected macaques [34], [46]–[50]. The most striking difference was the high expression of CD39 by Tregs in untreated patients compared to controls. CD39 is an ectonuclease that starts the process of hydrolysis of extracellular ATP into adenosine, which in turn, inhibits T cell proliferation and cytokine secretion [46]. Recent studies have shown that CD39 is mainly expressed by Tregs, although it is also found in a subset of FOXP3^−^ memory T cells that produce proinflammatory cytokines and are not suppressive [47]. Increased levels of CD39 by Tregs during HIV infection, as found by our group (Fig. 4C) and others [48], [49], may be important in HIV pathogenesis. Indeed, a CD39 blocking Ab abolished Treg suppression of cytokine production by Gag-stimulated CD8^+^ T cells and a gene polymorphism associated with down-modulated *CD39* expression is linked with slower progression to AIDS [49]. However, the role of CD39 during HIV infection is complex, like that of Tregs, as we have shown that blocking CD39 activity abolishes the capacity of Tregs to control HIV infection in non-Tregs [9]. CD39 could also be involved in Treg survival in the inflammatory environment created by HIV infection, as CD39-mediated degradation of ATP protects mouse Tregs from P2X7 receptor-mediated death [25], [50]. CD39 expression was significantly decreased after HAART, although its levels after >46 weeks of HAART remained higher than in normal controls. Mechanisms underlying CD39 upregulation on Tregs during HIV infection are not known. Exposure to HIV gp120 could play a role, as intracellular levels of cyclic AMP control CD39 expression [51] and signaling of Tregs by HIV gp120 has been shown to increase their intracellular cyclic AMP levels [52]. However, the existence of additional mechanisms is suggested by the fact that HAART-mediated control of HIV replication is not sufficient to completely normalize the levels of expression, and that CD39 expression did not correlate with patients' viral loads. Increased microbial translocation, which is not completely controlled by HAART, could also be involved, as TLR agonists such as flagellin induce CD39 expression [53].

The role of Treg in HIV pathogenesis remains uncertain, likely because Tregs, like many immune processes appear to behave as a double-edged sword. Nevertheless, whatever the complexity of their function, it is important to better understand Treg dynamics during chronic infection and how HAART affects these dynamics. Our comprehensive study of Treg frequency, absolute numbers and phenotype confirms that chronic HIV infection has a pronounced effect on the Treg frequency and phenotype. The circulating Treg compartment is relatively spared by HIV infection compared to other CD4^+^ subsets, likely because their homeostasis is changed. Our data also show that Tregs express normal or high levels of molecules associated with function, supporting the hypothesis that Tregs remain functional during chronic HIV infection. Importantly, we also show that suppressive HAART reverses the majority of the Treg qualitative and quantitative differences associated with chronic HIV infection.

## Materials and Methods

### Study subjects and clinical samples

Untreated HIV-1 infected subjects (n = 11) were co-enrolled in our study and in a clinical trial of tenofovir/emitricitabine 300/200 mg tablets plus 400/100 mg of lopinavir-ritonavir twice daily (combination of two reverse transcriptase inhibitors Tenofovir and Emtricitabine, with along with two protease inhibitors, lopinavir and ritonavir, the latter used as a pharmacologic booster), given at standard doses. The patients had no active opportunistic infections or cancer. The patients were either treatment naïve or had interrupted HAART for more than 6 months. None had a history of genotypic resistance to HAART. An initial sample (“wk0”) was obtained prior to HAART initiation. Blood samples were then obtained from patients at follow-up visits at wk2, 4, 8 and 24 on HAART. Another sample was obtained at a minimum of 46 weeks of continuous treatment (median number of weeks after HAART initiation: 51, range: 46–88). Adherence to HAART was scaled from poor (1) to excellent (4) based upon compliance with study visits, picking up medication on time, self-reported medication adherence and HIV viral load measurements demonstrating the expected response to therapy. In most analyses, we also included cells from 8 age-matched HIV-uninfected healthy controls, coming from a group of 12 (Table 1). The number of individuals/group included in each analysis is specified. Written informed consent was obtained from all subjects. This study was approved by the University of Cincinnati Institutional Review Board.

### Viral loads

Plasma VL was measured by reverse transcriptase-polymerase chain reaction (Ultrasensitive HIV RT-PCR: Roche, Basel, Switzerland). The threshold of detection was 50 HIV-1 copies/mL.

### Cell Isolation

At each visit, approximately 40 ml of blood was collected in vacutainer tubes coated with heparin (BD Pharmingen, San Jose, CA). Peripheral blood mononuclear cells (PBMCs) were isolated using Ficoll-Hypaque (GE Healthcare, UK) gradient centrifugation within 3 hours of collection. PBMCs were cryopreserved in liquid nitrogen and longitudinal samples were analyzed together.

### Treg Immunophenotyping

Thawed PBMC were immunophenotyped using multi-parameter flow cytometry (1×10^6^ cells/tube). Fluorochrome-labeled antibodies used for this study include anti-CD3 (SK7) PerCP-Cy5.5-, anti-CTLA4 (BNI3) APC-, anti-CD25 (MA251) PECy7-conjugated (BD Bioscience, San Diego, CA); anti-CD127 (eBioRDR5) eFluor450-, anti-FOXP3 (PCH101) PE-, anti-CD8 (RPA-T8) APC-eFluor780-, anti-Ki67 (B56) FITC-, anti-CD39 (eBioA1) FITC-conjugated (eBioscience, San Diego, CA); anti-CD4 (RPA-T4) A700-, anti-FOXP3 (259D) AF647-, anti-HLA-DR (LN3) Pacific Blue-, anti-CD45RA (HI100) PECy7-, anti-CD45RO (UCHL1) Pacific Blue-, anti-CD69 (FN50) PECy7-conjugated (Biolegend, San Diego, USA); anti-cyclin B (V152) AF-647- and anti-p24 (KC57) PE-conjugated (Beckman Coulter, Fullerton, CA); unconjugated anti-Bim (Cell Signaling Technology, Beverly, MA); anti-Bcl-2 (sc-509) AF-647 conjugated (Santa Cruz Biotechnology, Santa Cruz, CA), unconjugated anti-GARP (Plato-1, Alexis Biochemicals, San Diego, CA). GARP was conjugated using Zenon PE mIgG2b labeling kit (Invitrogen; Carlsbad, CA) following manufacturer's instructions. For detection of Bim, a secondary anti-rabbit Ab (Jackson ImmunoResearch Laboratories, West Grove, PA) was used.

Intracellular staining for FOXP3 (with the PCH101 clone and, in some experiments, the 259D clone) was performed using the eBioscience reagents and protocol. All antibodies were titrated for optimal detection of positive populations and mean fluorescent intensity (MFI) prior to use. All samples were fixed in 1% paraformaldehyde before acquisition on an LSRII flow cytometer (BD). Flow cytometry data were analyzed using the DIVA software (BD). We used a negative biological population for FOXP3 (CD3^−^CD4^−^ cells) as reference to set up the cut-off for FOXP3^+^ cells in CD3^+^CD4^+^ T cells, as previously described [17].

### Statistical Analysis

Statistical analysis was performed using SAS® Version 9.2 (Cary, NC). Comparisons of patients at wk0 versus controls were done using the Wilcoxon Rank Sum test with the exception of expression of Bim and Bcl-2 where control samples were matched with samples from infected patients. For these analyses, the Wilcoxon Signed Rank test for paired data was performed. Comparisons of patients at wk 46+ versus controls were also done using the Wilcoxon Rank Sum test. Each of these tests was conducted at the two-sided 5% level of significance.

To investigate changes at wk2 to wk46+ versus wk0 in HIV-infected patients, the change from baseline score for each time point was calculated. A mixed effect repeated measures analysis of variance was run with patient as a random effect and week as the fixed effect. To test whether there was a significant change from wk0 at each time point after HAART initiation, the least square mean for each week was tested versus wk0. The Tukey-Kramer p-value adjustment for multiple comparisons was used in the analysis. Data were log transformed prior to calculating change from baseline scores in order to meet the assumptions of normality and equal variance for the model. This approach is testing relative changes versus baseline. Results were considered statistically significant if the adjusted p-value was less or equal to 0.05.

To investigate the relationship among variables, the Spearman's Rank correlation coefficient was estimated and tested for being significantly different from 0. This test was conducted at the two-sided 5% level of significance.

## Supporting Information

Figure S1 **Representative gating strategy to characterize Tregs.** PBMC from HIV-infected patients and uninfected subjects were purified and stained with anti-CD3, anti-CD4 and anti-FOXP3. Dead cells were excluded based on SS and FS. CD3^+^CD4^−^ cells were used as a reference for FOXP3^+^ cell detection in CD3^+^CD4^+^ cells.
(TIFF)
