# Supplementary figures and images for: Frequency of Circulating Regulatory T Cells Increases during Chronic HIV Infection and Is Largely Controlled by Highly Active Antiretroviral Therapy
Source: PLoS One. 2011 Dec 5;6(12):e28118. doi: 10.1371/journal.pone.0028118 (PMC3230597; doi:10.1371/journal.pone.0028118)

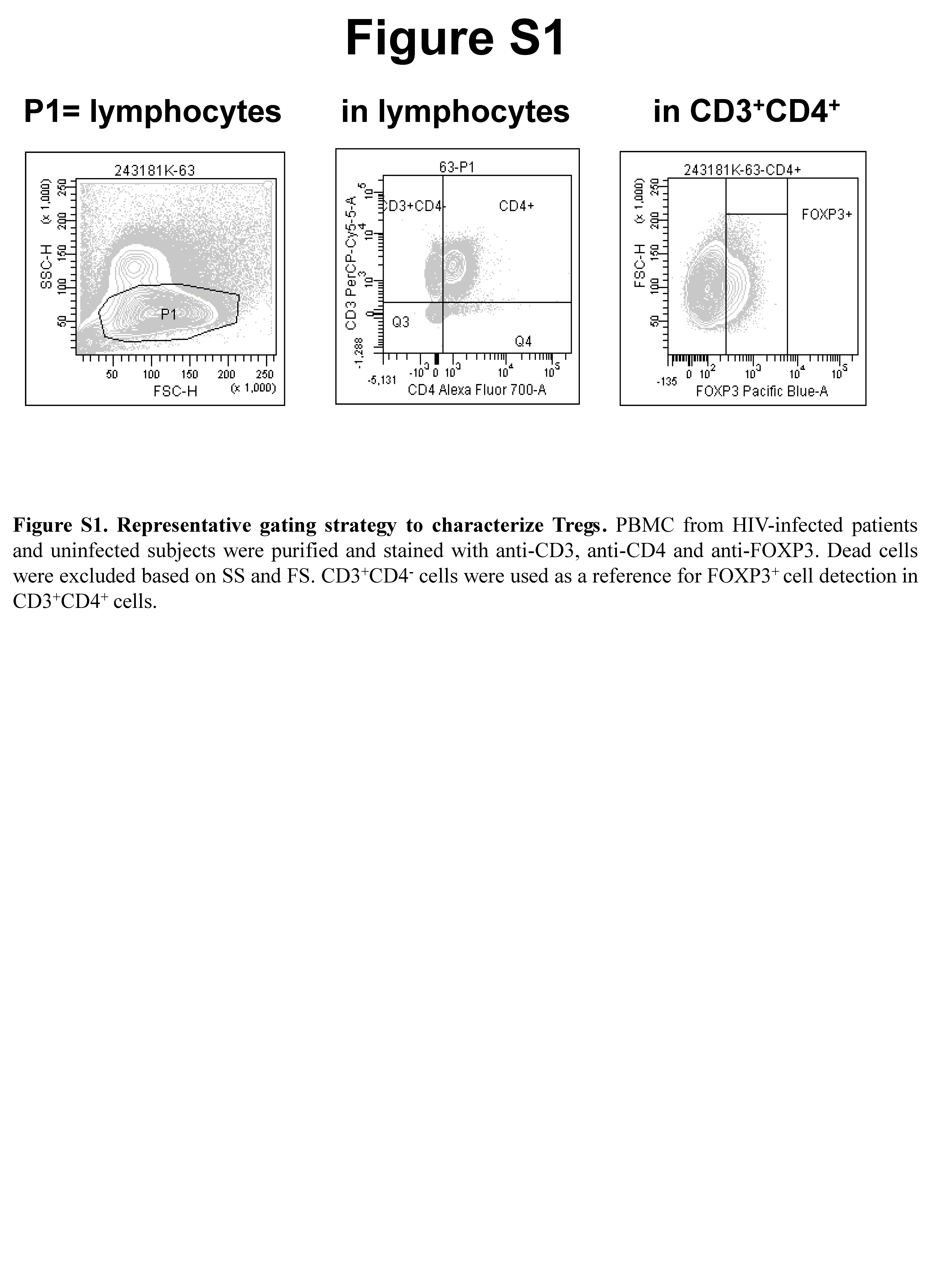

Supplement: Figure S1 — Representative gating strategy to characterize Tregs. PBMC from HIV-infected patients and uninfected subjects were purified and stained with anti-CD3, anti-CD4 and anti-FOXP3. Dead cells were excluded based on SS and FS. CD3+CD4− cells were used as a reference for FOXP3+ cell detection in CD3+CD4+ cells. (TIFF) [file pone.0028118.s001.tiff]
